# FGF1 and FGF19 reverse diabetes by suppression of the hypothalamic–pituitary–adrenal axis

**DOI:** 10.1038/ncomms7980

**Published:** 2015-04-28

**Authors:** Rachel J. Perry, Sangwon Lee, Lie Ma, Dongyan Zhang, Joseph Schlessinger, Gerald I. Shulman

**Affiliations:** 1 Howard Hughes Medical Institute, Yale University School of Medicine, New Haven, Connecticut 06536-8012, USA; 2 Department of Internal Medicine, Yale University School of Medicine, New Haven, Connecticut 06536-8012, USA; 3 Department of Cellular & Molecular Physiology, Yale University School of Medicine, New Haven, Connecticut 06536-8012, USA; 4 Department of Pharmacology, Yale University School of Medicine, New Haven, Connecticut, USA

## Abstract

Fibroblast growth factor-1 (FGF1) and FGF19 have been shown to improve glucose metabolism in diabetic rodents, but how this occurs is unknown. Here to investigate the mechanism of action of these growth factors, we perform intracerebroventricular (ICV) injections of recombinant FGF1 or FGF19 in an awake rat model of type 1 diabetes (T1D) and measure rates of whole-body lipolysis, hepatic acetyl CoA content, pyruvate carboxylase activity and hepatic glucose production. We show that ICV injection of FGF19 or FGF1 leads to a ∼60% reduction in hepatic glucose production, hepatic acetyl CoA content and whole-body lipolysis, which results from decreases in plasma ACTH and corticosterone concentrations. These effects are abrogated by an intra-arterial infusion of corticosterone. Taken together these studies identify suppression of the HPA axis and ensuing reductions in hepatic acetyl CoA content as a common mechanism responsible for mediating the acute, insulin-independent, glucose-lowering effects of FGF1 and FGF19 in rodents with poorly controlled T1D.

## Abstract (web summary)

Fibroblast growth factor (FGF) family proteins have anti-diabetic effects, but how they work is currently unclear. Here the authors show that injections of FGF1 or FGF19 into the brain of diabetic rats alter glucose and lipid homeostasis by suppressing activity of the hypothalamic-pituitary-adrenal signalling axis.

## Graphical abstract

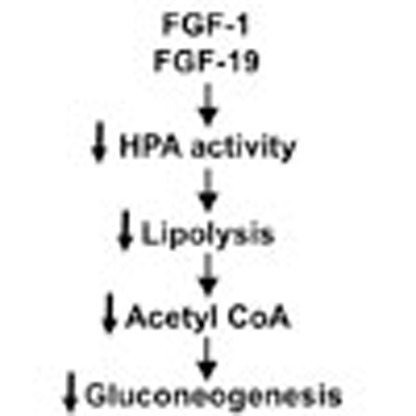

Recent studies have generated a great deal of interest in the insulin-independent glucose-lowering effects of fibroblast growth factor-19 (FGF19) and FGF1 in diabetic rodents and their potential application as novel anti-diabetic therapies^1^,^2^,^3^,^4^; however, little is known regarding the mechanism by which these factors decrease plasma glucose concentrations. FGF19 is an atypical FGF which together with FGF21 and FGF23 is designated an endocrine FGF. It is now well established that members of the FGF family of growth factors mediate their cellular responses by binding together and acting in concert with heparin sulfate proteoglycans to activate the four receptor tyrosine kinases designated FGFR1–4. The endocrine members of the FGF family, designated FGF19, -21 and -23, mediate their endocrine functions by binding selectively to FGFR1c, FGFR2c, FGFR3c or FGFR4 together with β-Klotho or α-Klotho, respectively, to mediate their multiple endocrine functions^1^,^2^,^3^. FGF19 has been proposed to play diverse physiological roles, modulating development, metabolism, neuronal signalling, atherosclerosis and carcinoma cell proliferation^5^. The rodent homologue of FGF19 is designated FGF15, and because FGF15 and FGF19 activate both human and rodent FGFRs, application of exogenous FGF19 provides a model system to study the effects of exogenous FGF19 on *in vivo* metabolism in rodents^6^,^7^.

Interestingly both intraperitoneal injection of FGF19 and overexpression of FGF19 in transgenic mice reversed diet-induced insulin resistance and hyperglycemia in an insulin-independent manner^8^,^9^,^10^. Recently, Morton *et al.*^1^ have reported that a single intracerebroventricular (ICV) injection of FGF19 improved glucose homeostasis in leptin-deficient *ob/ob* mice without affecting insulin secretion or whole-body insulin sensitivity. This finding, which was independent of changes in body composition or food intake, implies a central action of FGF19 to acutely lower plasma glucose concentrations.

In contrast to FGF19, FGF1 is a prototype FGF, which has been well studied for its roles in development, including angiogenesis and morphogenesis^11^. Surprisingly FGF1 has also recently been shown to lower plasma glucose concentrations in diabetic mice^4^, while mice genetically deficient in FGF1 exhibit insulin resistance on a high fat diet^12^. However the molecular mechanism by which FGF1 lowers blood glucose is also unknown.

In this regard we have recently demonstrated that increased hypothalamic–pituitary–adrenal (HPA) axis activity, due to acquired leptin deficiency, is critical in promoting hyperglycemia in poorly controlled type 1 diabetes (T1D) and T2D. This study found that increased plasma corticosterone concentrations led to increased rates of lipolysis and gluconeogenesis through increases in the allosteric activation of pyruvate carboxylase (PC) by increases in hepatic acetyl CoA content and increased glycerol turnover^13^. Similar to the effect of leptin, we hypothesized that ICV administration of FGF1 or FGF19 in a streptozotocin-induced rat model of T1D might lower plasma glucose concentrations and normalize rates of hepatic glucose production by suppressing lipolysis through reductions in activation of the HPA axis. Here we show that FGF1 and FGF19 indeed suppress adrenocorticotropic hormone (ACTH) and corticosterone, leading to reductions in whole-body lipolysis, hepatic acetyl CoA content and PC activity, thereby suppressing hepatic glucose production. These data thus identify a common mechanism for the action of FGF1 and FGF19 to ameliorate hyperglycemia in poorly controlled diabetes by suppression of the HPA axis.

## Results

### Glucocorticoid suppression reduces lipolysis and HGP

**Figure 1:**
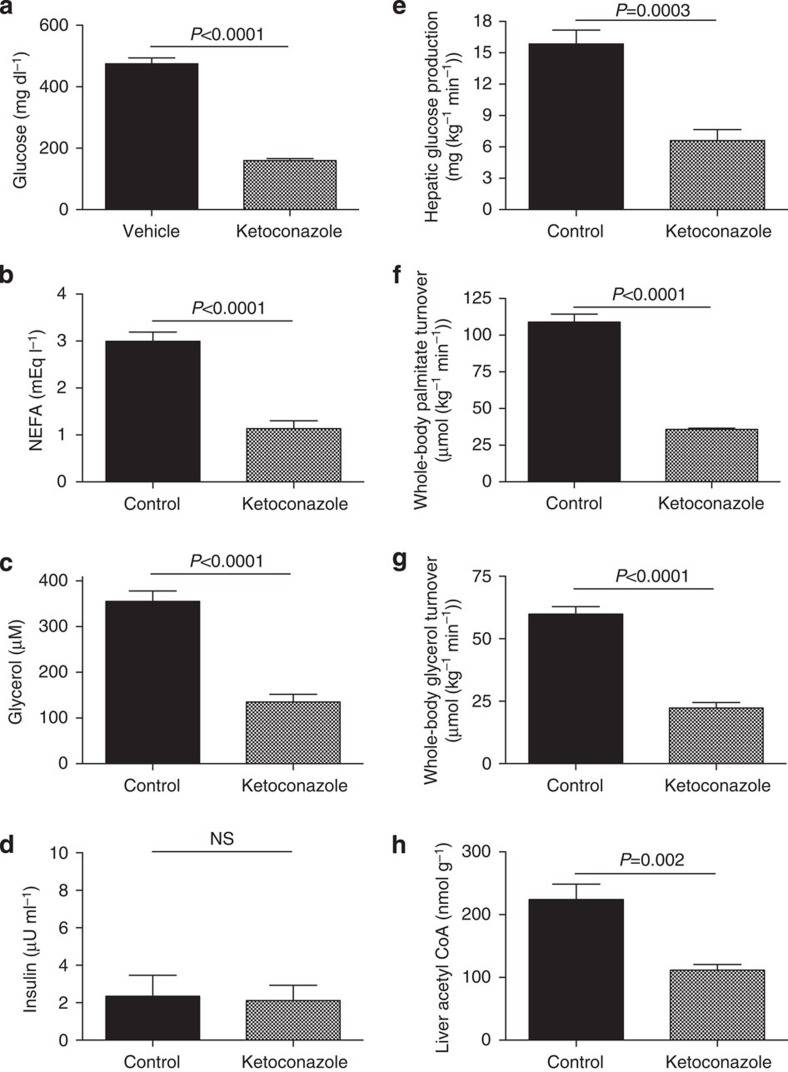
Treatment with a glucocorticoid receptor antagonist reduces lipolysis and plasma glucose concentrations in T1D rats. (**a**–**d**) Fasting plasma glucose, NEFA, glycerol and insulin concentrations. (**e**) Hepatic glucose production. (**f**–**g**) Whole-body palmitate and glycerol turnover. (**h**) Liver acetyl CoA. Data are representative of mean±s.e.m. of *n*=6 per group, with comparisons by the two-tailed unpaired Student's *t*-test.

Suppression of glucocorticoid release with ketoconazole resulted in reductions in plasma glucose, non-esterified fatty acid (NEFA) and glycerol concentrations without any change in plasma insulin concentrations (Fig. 1a-d). These reductions in plasma NEFA and glycerol concentrations could be attributed to reductions in whole-body rates of lipolysis as reflected by 60% reductions in rates of glycerol and palmitate turnover and were associated with a similar reduction in hepatic glucose production (Fig. 1e-g). Acetyl CoA, a potent activator of PC^14^, was also reduced by 50% with ketoconazole treatment (Fig. 1h), again demonstrating a key role for glucocorticoid-induced lipolysis and increased hepatic acetyl CoA in mediating hyperglycemia in a rat model of poorly controlled T1D.

### FGF19 suppresses HGP by reducing hepatic acetyl CoA

**Figure 2:**
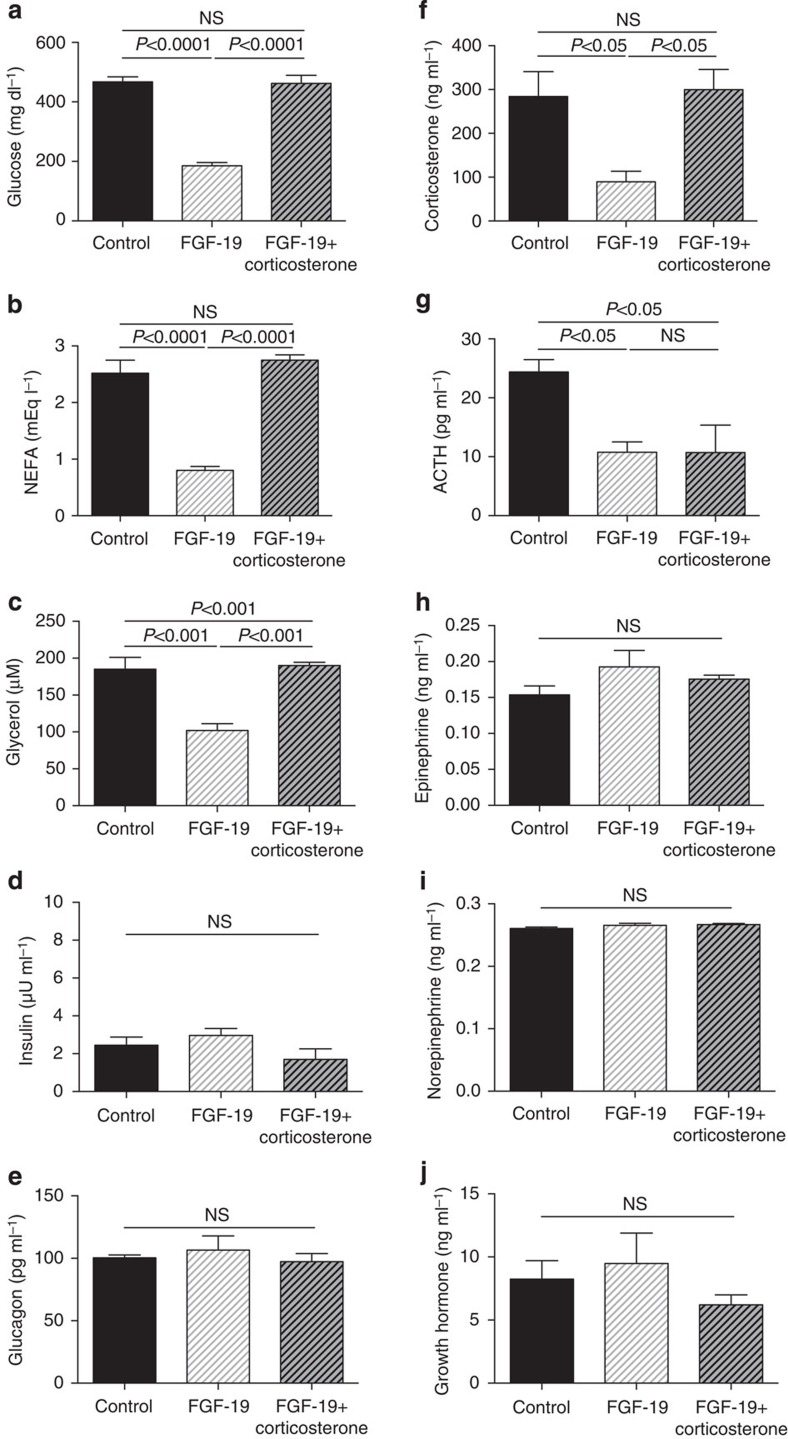
FGF19 (30 μg) corrects hyperglycemia in T1D rats by suppression of the hypothalamic–pituitary–adrenal axis. (**a**–**c**) Plasma glucose, NEFA and glycerol concentrations. (**d**–**e**) Fasting plasma insulin and glucagon concentrations. (**f**–**j**) Plasma corticosterone, ACTH, epinephrine, norepinephrine and growth hormone concentrations. Data are representative of mean±s.e.m. of *n*=6 per group, with comparisons by analysis of variance with Bonferroni’s multiple comparisons test.

**Figure 3:**
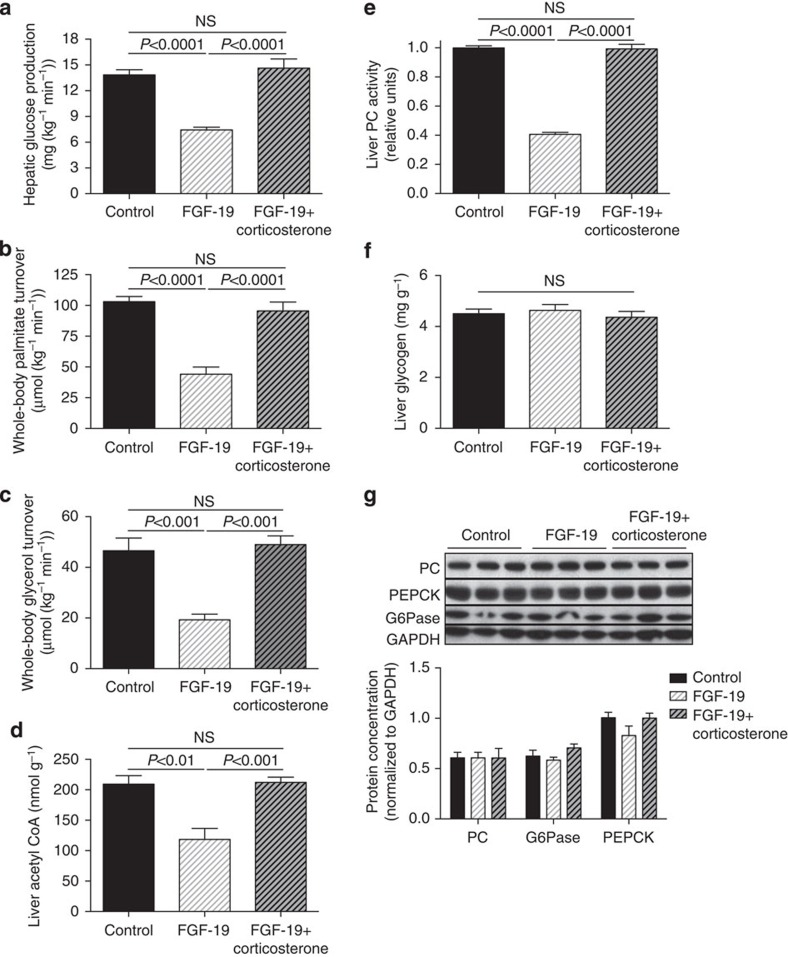
FGF19 (30 μg) corrects hyperglycemia in T1D rats by reducing lipolysis and hepatic acetyl CoA content. (**a**) Hepatic glucose production. (**b**,**c**) Whole-body palmitate and glycerol turnover. (**d**) Hepatic acetyl CoA concentrations. (**e**) Liver pyruvate carboxylase activity. (**f**) Liver glycogen content. (**g**) Liver gluconeogenic protein concentrations. Data are representative of mean±s.e.m. of *n*=6 per group, with comparisons by analysis of variance with Bonferroni’s multiple comparisons test.

We next examined the hypothesis that ICV injection of FGF19 would improve plasma glucose concentrations in a severely hyperglycemic, insulinopenic rat model of T1D and found that plasma glucose, NEFA and glycerol concentrations were reduced markedly within 6 h following injection of 30 μg ICV FGF19 (Fig. 2a–c). This reduction in hyperglycemia was associated with a 70% reduction in plasma corticosterone and ACTH concentrations but occurred independently of any change in plasma insulin, glucagon, epinephrine, norepinephrine or growth hormone concentrations (Fig. 2d–j). Suppression of corticosterone was associated with reductions in hepatic glucose production and in whole-body lipolysis as reflected by reductions in glycerol and palmitate turnover (Fig. 3a–c). Reduced HGP in FGF19-treated rats was associated with 50–60% reductions in hepatic PC activity as well as hepatic acetyl CoA content (Fig. 3d,e). In contrast this perturbation had no effect on liver (Fig. 3f) or muscle glycogen content (4.5±0.3 versus 4.7±0.2 versus 4.5±0.2 mg g^−1^), or on hepatic gluconeogenic protein expression (Fig. 3g, Supplementary Fig. 1),

Taken together these data suggest that suppression of the HPA axis may contribute to FGF19’s glucose-lowering effect in T1D rats. To further test this hypothesis, we performed a 6-h intra-arterial infusion of corticosterone immediately following FGF19 treatment. Restoring plasma corticosterone concentrations in FGF19-treated T1D rats to those measured in untreated T1D rats totally abrogated the glucose-lowering effects of FGF19 as well as its effects on plasma glucose, lactate, NEFA, glycerol concentrations and rates of palmitate and glycerol turnover (Figs 2, 3). As predicted by these data, plasma corticosterone concentrations were strong predictors of plasma glucose (*r*^2^=0.63), palmitate (*r*^2^=0.81) and glycerol turnover (*r*^2^=0.84), with both palmitate and glycerol turnover correlating tightly with rates of hepatic glucose production (*r*^2^=0.60 and 0.61, respectively).

**Figure 4:**
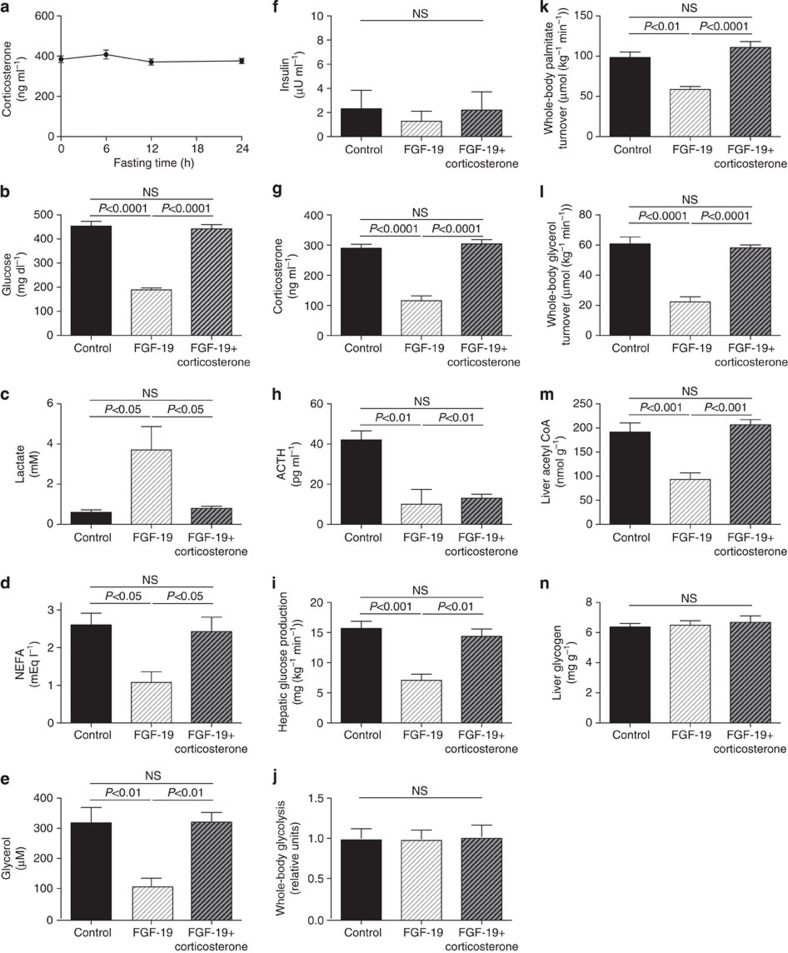
Low-dose FGF19 (10 μg) lowers plasma glucose concentrations in T1D rats by suppressing the HPA axis. (**a**) Plasma corticosterone concentrations after 0–24 h of fasting in T1D rats. (**b**–**e**) Fasting plasma glucose, lactate, NEFA and glycerol concentrations. (**f**–**h**) Plasma insulin, corticosterone and ACTH concentrations. (**i**) Hepatic glucose production. (**j**) Whole-body glycolysis. (**k**,**l**) Whole-body palmitate and glycerol turnover. (**m**) Liver acetyl CoA. (**n**) Liver glycogen. Data are representative of mean±s.e.m. of *n*=6 per group, with comparisons by analysis of variance with Bonferroni’s multiple comparisons test.

To further test the hypothesis that the effects of FGF19 on glycaemia in T1D rodents are due to suppression of the HPA axis and independent of the length of the fasting time (18 h) (Fig. 4a), we repeated these studies in rats with food withdrawn for only 6 h and treated these T1D animals with a three-fold lower ICV dose of FGF19 (10 μg). These studies confirmed the previous observations, with similar reductions in plasma glucose, corticosterone and ACTH concentrations as well as rates of hepatic glucose production, lipolysis and hepatic acetyl CoA content observed in rats treated with low-dose FGF19 and fasted for 6 h. Additionally FGF19 treatment raised plasma lactate concentrations as previously described^1^ but did not alter rates of whole-body glycolysis. These effects were all abrogated by an intra-arterial infusion of corticosterone to match plasma concentrations of untreated T1D rats (Fig. 4b–n).

### FGF19 suppresses HGP by reducing hepatic acetyl CoA

**Figure 5:**
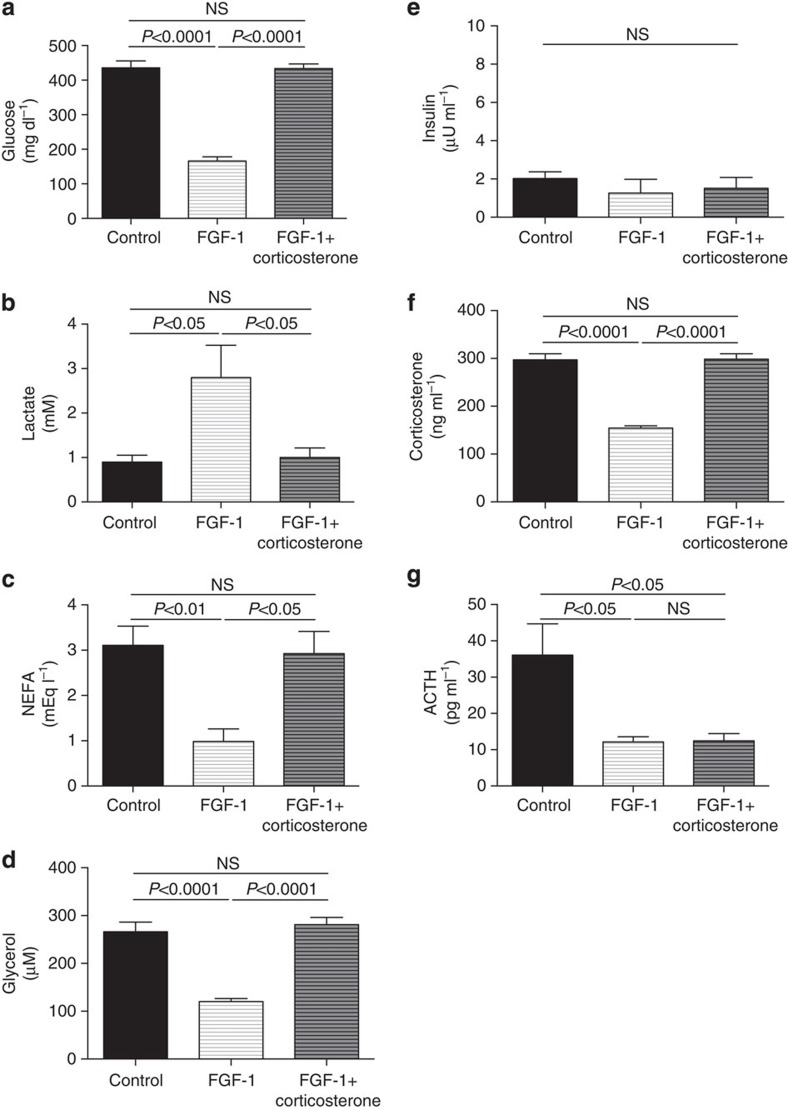
FGF1 (10 μg) reverses hyperglycemia in type 1 diabetic rats by suppressing the HPA axis. (**a**–**d**) Plasma glucose, lactate, NEFA and glycerol concentrations. (**e**–**g**) Plasma insulin, corticosterone and ACTH concentrations. Data are representative of mean±s.e.m. of *n*=6 per group, with comparisons by analysis of variance with Bonferroni’s multiple comparisons test.

**Figure 6:**
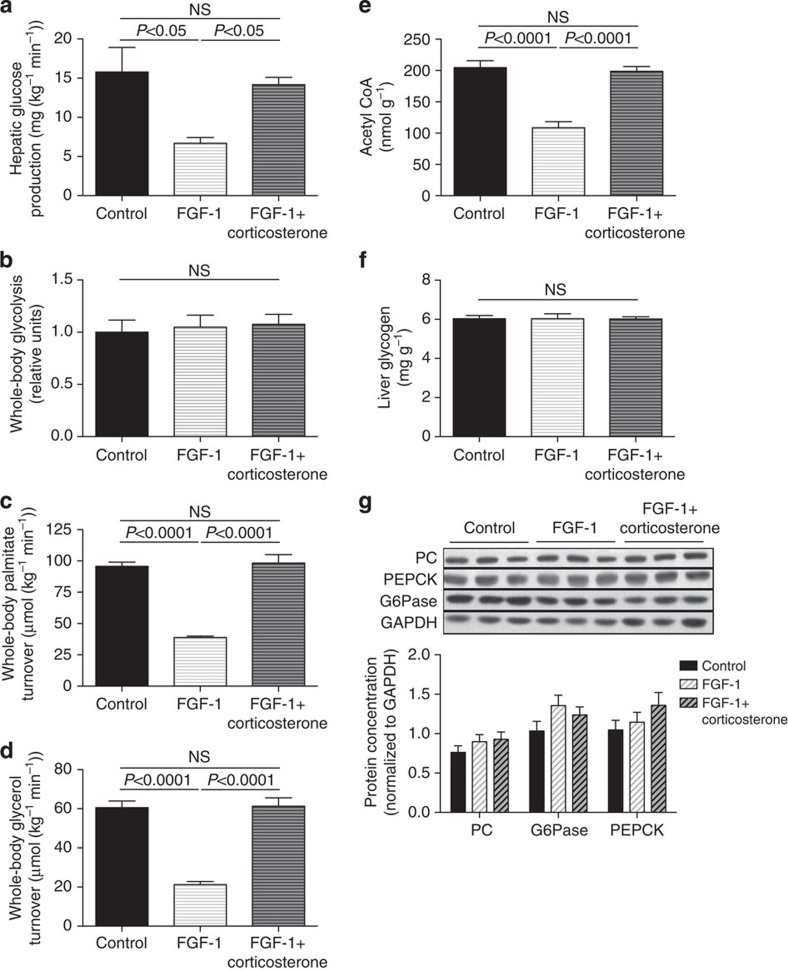
FGF1 (10 μg) reverses hyperglycemia in type 1 diabetic rats by suppressing the HPA axis, thereby reducing lipolysis and hepatic glucose production. (**a**) Hepatic glucose production. (**b**) Whole-body glycolysis. (**c**–**d**) Whole-body palmitate and glycerol turnover. (**e**) Liver acetyl CoA. (**f**) Hepatic glycogen content. (**g**) Liver gluconeogenic protein expression. Data are representative of mean±s.e.m. of *n*=6 per group, with comparisons by analysis of variance with Bonferroni’s multiple comparisons test.

Next we hypothesized that FGF1 may have similar effects to suppress the HPA axis resulting in lower rates of whole-body lipolysis, hepatic acetyl CoA and hepatic glucose production in insulinopenic T1D rodents. To test this hypothesis, we treated 6-h fasted T1D rats with 10 μg ICV FGF1 and observed >50% reductions in plasma glucose, NEFA and glycerol concentrations and increased plasma lactate concentrations despite unchanged plasma insulin concentrations (Fig. 5a–e). These FGF1-induced effects were associated with marked suppression of the HPA axis, indicated by 50–60% reductions in plasma corticosterone and ACTH concentrations (Fig. 5f–g). Similar to the FGF19 infusion studies, the antihyperglycemic effect of FGF1 could be attributed to reductions in rates of whole-body lipolysis, liver acetyl CoA and hepatic glucose production without any change in rates of whole-body glycolysis (Fig. 6a–e). Akin to the glucose-lowering effects of FGF19, all of these glucose-lowering effects of FGF1 were abrogated by an intra-arterial corticosterone infusion to match plasma corticosterone concentrations to those of the untreated T1D rats and were independent of any alterations in liver (Fig. 6f) or muscle (5.8±0.2 versus 5.9±0.3 versus 5.9±0.1 mg g^−1^) glycogen content or hepatic gluconeogenic protein expression (Fig. 6g, Supplementary Fig. 2).

### Low-dose insulin suppresses lipolysis and HGP

**Figure 7:**
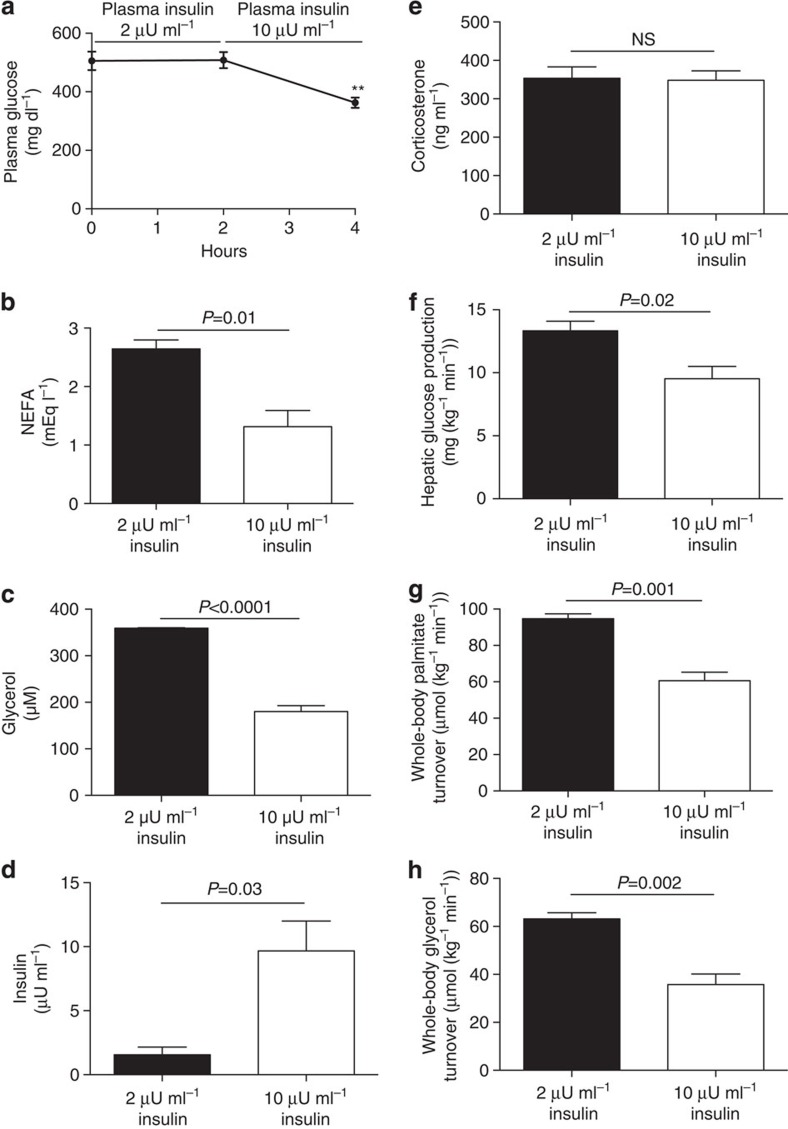
Increasing plasma insulin concentrations in T1D rats from 2 to 10 μU ml^−1^ suppresses lipolysis. (**a**–**d**) Plasma glucose, NEFA, glycerol and insulin concentrations. (**e**) Plasma corticosterone. (**f**) Hepatic glucose production. (**g**,**h**) Whole-body palmitate and glycerol turnover. Data are representative of mean±s.e.m. of *n*=6 rats, with comparisons by the two-tailed paired Student’s *t*-test.

We next studied T1D rats both before and after infusion of a low-dose insulin infusion to raise plasma insulin concentrations from 2 to 10 μU ml^−1^ (Fig. 7a). This small increase in plasma insulin concentrations resulted in 50% reductions in plasma NEFA and glycerol concentrations, which were associated with 30% reductions in rates of lipolysis and hepatic glucose production despite unchanged hypercorticosteronemia (Fig. 7b–h).

## Discussion

In this study, we applied a novel liquid chromatography/tandem mass spectrometry technique to measure hepatic acetyl CoA content in *in situ* freeze-clamped liver within 10 s of intravenous euthanasia in chronically arterio-venous catheterized, awake rats. The advantage of studying this model is that tracer infusions and blood sampling can be performed in free-ranging animals with minimal stress, which would impact HPA activity. These measurements were combined with measurements of whole-body rates of lipolysis, assessed by rates of [1,1,2,3,3-^2^H_5_] glycerol and [U-^13^C] palmitate turnover, combined with rates of hepatic glucose production assessed by [3-^3^H] glucose turnover. Using this comprehensive metabolic flux approach, we first examined the hypothesis that hypercorticosteronemia is necessary and sufficient to maintain hyperglycemia in an awake rat model of poorly controlled T1D. To this end, we treated T1D rats with a potent inhibitor of glucocorticoid biosynthesis, ketoconazole. Consistent with our previous results using mifepristone^14^, a potent glucocorticoid receptor antagonist, ketoconazole treatment acutely normalized lipolysis, hepatic acetyl CoA concentrations and hepatic glucose production without affecting plasma insulin concentrations.

We next examined the hypothesis that FGF1 and FGF19 may act through a similar mechanism, suppressing the HPA axis and reducing lipolysis, hepatic acetyl CoA content and hepatic glucose production, and our findings are concordant with this hypothesis. We also demonstrate an increase in plasma lactate concentrations without any change in rates of whole-body glycolysis or changes in liver or muscle glycogen content, suggesting an alternative mechanism for the glucose-lowering effects of FGF19 than that posited by Morton *et al.*^1^, who suggested that ICV FGF19 treatment promotes increased glycolysis in peripheral tissues resulting in the observed increase in plasma lactate concentration. We tested this hypothesis by directly measuring whole-body glycolysis but did not observe any difference in rates of glycolysis between groups, suggesting that the observed effects of ICV FGF19 to increase plasma lactate concentrations can mostly be attributed to reduced conversion of lactate to glucose, due to decreased PC activity. Consistent with this hypothesis, we observed that these effects of FGF19 to increase plasma lactate concentrations were abrogated by an intra-arterial infusion of corticosterone, which led to increased lipolysis, increased hepatic acetyl CoA content and increased PC activity without impacting rates of glycolysis.

These data demonstrate that ICV infusion of FGF19 or FGF1 promotes similar, insulin-independent plasma glucose-lowering effects in T1D rodents acutely through suppression of the HPA axis resulting in reduced rates of lipolysis, hepatic acetyl CoA content, PC activity and hepatic glucose production. In addition to its acute glucose-lowering effects on the HPA axis, chronic FGF19 treatment has also been shown to result in increased energy expenditure, reduced food intake and lower body weight^2^,^3^, which also likely contributes to its efficacy at improving glycaemia in chronically treated rodent models of obesity and diabetes. In the absence of a definitive molecular mechanism of the action of FGF1 and FGF19, indirect actions to lower glycaemia cannot be definitively ruled out as well as whether systemic injections of these proteins will lead to similar effects on the HPA axis since it is unknown if these proteins can cross the blood–brain barrier.

It is noteworthy that FGF1 and FGF19 activate a different complement of FGFRs. While FGF1 stimulates the activities of both the ‘b’ and ‘c’ isoforms of FGF1, 2 and 3, FGF19 stimulates only the activities of the ’c’ isoforms of FGF1, 2 and 3 in β-Klotho dependent manner. Moreover, both FGF1 and FGF19 also stimulate the activation of FGFR4, a member of the FGFR family lacking the ‘b’ isoforms. While all members of the FGFR family as well as β-Klotho are expressed in the brain, the exact complement of FGFRs that are responsible for mediating the cascade of signals that are initiated in the CNS leading to glucose lowering in rodents remain to be identified.

Our study, demonstrating a critical role for hypercorticosteronemia in mediating increased lipolysis and hyperglycemia in a rodent model of T1D, stands in contrast to previous studies in which adrenalectomy was demonstrated to have little to no effect on hyperglycemia in streptozotocin-treated rats^15^,^16^. We hypothesized that given the exquisite sensitivity of the adipocyte to insulin, the higher plasma insulin concentrations (10–55 μU ml^−1^) measured in these previous studies^15^,^16^, as compared with ∼2 μU ml^−1^ in our study, may be sufficient to trump hypercorticosteronemia and suppress lipolysis and hepatic glucose production in the prior studies. In concert with this hypothesis, our data demonstrate that relatively low plasma concentrations of insulin (∼10 μU ml^−1^), which are insufficient to maintain euglycemia in an insulinopenic rat model of T1D, are sufficient to suppress lipolysis in the presence of hypercorticosteronemia and offer an explanation as to the absence of an effect of adrenalectomy on glycaemia in these previous studies^15^,^16^.

In summary, these studies identify FGF19 and FGF1 suppression of HPA axis-mediated lipolysis, resulting in reductions in hepatic acetyl CoA content, PC activity and glycerol turnover, as the major and common mechanism responsible for the insulin-independent effects of FGF19 and FGF1 to acutely reduce hepatic glucose production and hyperglycemia in a rodent model of poorly controlled T1D.

## Methods

### Animals

All protocols were approved by the Yale University Animal Care and Use Committee. Normal male Sprague-Dawley rats (9-weeks old, ∼300 g) were obtained from Charles River and were housed individually under 12 h light/12 h dark conditions. Rats underwent surgery to place catheters in the third ventricle of the brain, the carotid artery and the jugular vein. After recovery (∼5 days and recovery of pre-surgery body weight), diabetes was induced by injection of streptozotocin (65 mg kg^−1^). Hyperglycemia was confirmed by measuring blood glucose concentrations using an AlphaTrak glucometer 24 h after injection of streptozotocin, and rats with plasma glucose >300 mg dl^−1^ were studied. Rats with fasting plasma insulin concentrations ≥10 μU ml^−1^ were later excluded from analysis. All rats were fasted overnight prior to study. No specific randomization method was used; however, we confirmed that rats in all groups had body weights within 20 g of each other before the start of the FGF treatment experiment. For practical reasons, the investigators were not blinded during these studies.

### Preparation of recombinant FGF19 and FGF1

Human FGF19 (amino acid 25–216) was expressed in *Escherichia coli* as an insoluble fraction, refolded and purified as previously described^17^. Human FGF1 (amino acid 1–155) was expressed in *E. coli* and purified from the soluble cell lysate by heparin affinity and size exclusion chromatography. The purified protein was flash-frozen and stored at −80 °C until further use.

### ICV FGF19 and corticosterone treatment

At 0800 hours, rats underwent an ICV injection of FGF19 (30 μg, high dose; 10 μg, low dose) or saline vehicle. A subset of FGF19-treated rats received a continuous intra-arterial infusion of corticosterone for 6 h (12.5 mg kg^−1^ total). All rats underwent studies of glucose and lipid turnover and were killed 6 h after treatment with FGF19 or vehicle.

### ICV FGF1 and corticosterone treatment

At 0800 hours, rats were treated with an ICV injection of FGF1 (10 μg) or saline vehicle and a 6 h fast was begun at that time. A subset of FGF1-treated rats received a continuous intra-arterial infusion of corticosterone for 6 h (12.5 mg kg^−1^ total). For the last 2 h of the study, rats underwent 120 min infusions of glucose, fatty acid and glycerol tracers to measure the turnover of each metabolite as described below. Rats were killed 6 h after treatment with FGF1 or vehicle.

### Insulin infusions

Overnight fasted type 1 diabetic rats underwent 120 min studies of glucose, fatty acid and glycerol turnover as described below. After collecting of a blood sample (400 μl), they subsequently received an arterial infusion of regular insulin (0.4 mU kg^−1^ min^−1^) for 120 min. A second blood sample (1 ml) was taken and the rats were euthanized.

### Ketoconazole treatment

Overnight fasted type 1 diabetic rats were treated with ketoconazole (175 mg kg^−1^ in PEG 400) by oral gavage. Two hours later, they underwent studies of glucose, fatty acid and glycerol turnover as described below, and euthanized at the end of the 120 min infusions.

### Substrate turnover studies

To measure hepatic gluconeogenesis, whole-body palmitate and glycerol turnover, we performed a 120 min infusion of [3-^3^H] glucose (0.05 μCi/min), [1,1,2,2,3-d_5_] glycerol (1.5 μmol kg^−1^ min^−1^) and [U-^13^C] potassium palmitate (30 mg kg^−1^ min^−1^) beginning 4 h after FGF19 or vehicle treatment. After 120 min of infusion, a blood sample was drawn from the venous catheter and rats were euthanized with intravenous pentobarbital and livers freeze-clamped in liquid nitrogen, then stored at −80 °C for further analysis.

Hepatic gluconeogenesis was measured by determining [^3^H] glucose-specific activity using a scintillation counter and assuming that all measured glucose production was from gluconeogenesis in these overnight fasted, glycogen-depleted rats and that 90% of the total gluconeogenesis was from the liver, based on our previous findings in T1D rats^13^. Whole-body glycolysis was calculated using [^3^H] glucose-specific activity^18^, and normalized to the mean glycolysis rate in T1D control rats. Whole-body glycerol and palmitate turnover were calculated based on plasma glycerol and palmitate enrichment measured by gas chromatography/mass spectrometry as we have described^13^.

### Plasma analysis

Plasma insulin (Mercodia), corticosterone (Abcam), ACTH (MyBioSource), growth hormone (Life Technologies), epinephrine (Abnova), norepinephrine (Abnova) and glucagon (Sigma) concentrations were measured by ELISA. Plasma corticosterone and ACTH concentrations were measured in all animals at 1400 hours. Plasma NEFA concentrations were measured using a Wako kit, glycerol concentrations were measured by gas chromatography/mass spectrometry, and lactate concentrations were measured by COBAS.

### Tissue analysis

Hepatic acetyl CoA content was measured by liquid chromatography/mass spectrometry/mass spectrometry as we described earlier^19^. Liver PC activity was measured in all samples as previously described^20^, matching acetyl CoA concentrations in the assay to those measured *in vivo* (200 nmol ml^−1^ for the control and FGF19+corticosterone-treated groups and 100 nmol ml^−1^ for the FGF19-treated rats). Liver and gastrocnemius glycogen content was measured using the method by Passoneau and Lauderdale^21^. Hepatic PC, glucose-6-phosphatase and cytosolic phosphoenolpyruvate carboxykinase protein content was measured by Western blot^22^ and normalized to GAPDH content. Antibodies to each protein were obtained from Santa Cruz (catalogue numbers sc-46230, sc-33841, sc-377136 and sc-20357, respectively). Dilutions of 1:200 were used for PC, glucose-6-phosphatase and phosphoenolpyruvate carboxykinase, and a 1:5,000 dilution was used for GAPDH.

### Statistical analysis

The two-tailed Student’s *t*-test was used to compare two groups, with either the paired or unpaired *t*-test selected as denoted in the figure legends depending on whether the same animals were used in both groups (Fig. 7) or whether the groups represented different rats (all other figures). When three groups were compared (Figs 2, 3, 4, 5, 6, Supplementary Figs S1–S2), the one-way analysis of variance with Bonferroni’s multiple comparisons test was used for comparison. Prism software was used to perform these statistical tests and to confirm that the variance was similar within each group compared. In each figure, the data are presented as the mean±s.e.m. of *n*=6 rats per group. This group size was selected to detect moderate-to-large differences based on a power calculation.

## Additional information

**How to cite this article**: Perry, R.J. *et al.* FGF1 and FGF19 reverse diabetes by suppression of thehypothalamic-pituitary-adrenal axis. *Nat. Commun.* 6:6980 doi: 10.1038/ncomms7980 (2015).

## Supplementary Material

Supplementary Information Supplementary Figures 1-2
