# Supplementary material for: FGF1 and FGF19 reverse diabetes by suppression of the hypothalamic–pituitary–adrenal axis
Source: Nat Commun. 2015 Apr 28;6:6980. doi: 10.1038/ncomms7980 (PMC4413509; doi:10.1038/ncomms7980)
Supplement: Supplementary Information — Supplementary Figures 1-2 [file ncomms7980-s1.pdf]

Supplementary Figure 1

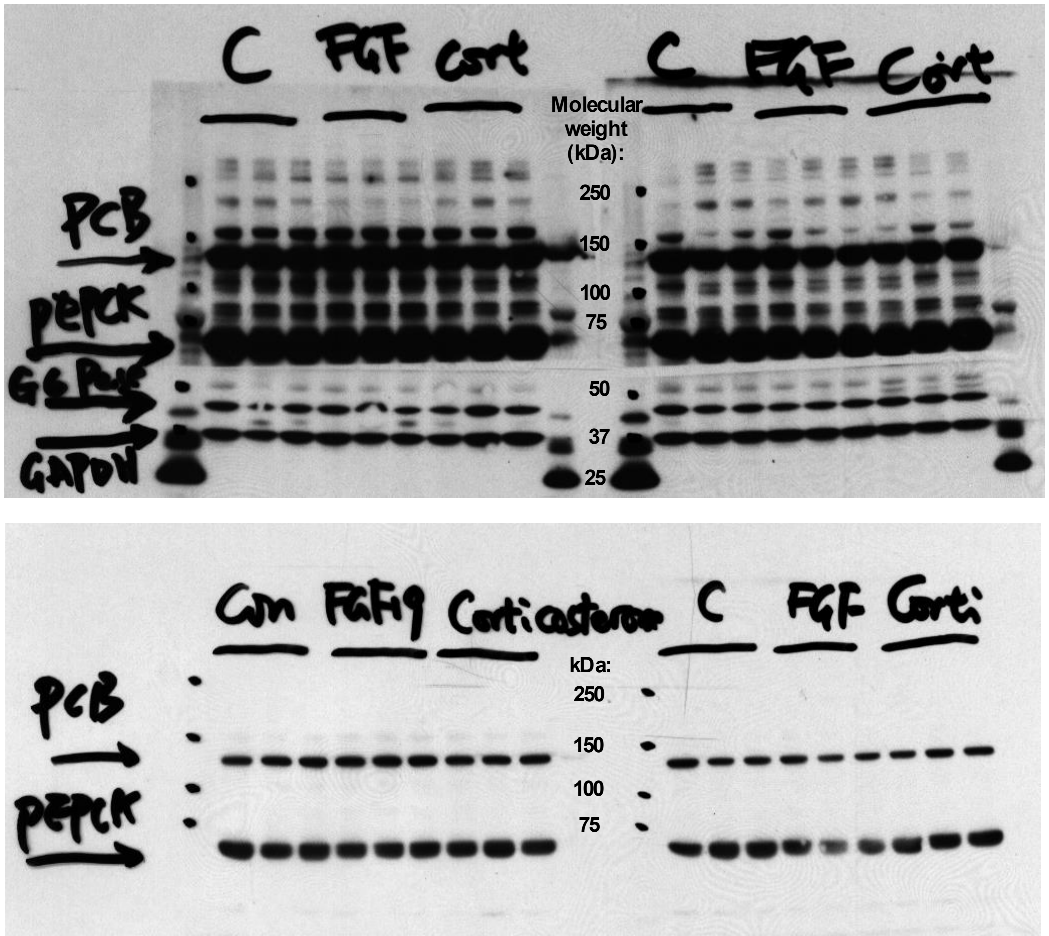

Supplementary Figure 1. Uncut Western blots demonstrating that FGF19 (30 µg) does not affect liver gluconeogenic protein expression. PCB, pyruvate carboxylase isoform B; PEPCK, cytosolic phosphoenolpyruvate carboxykinase; G6Pase, glucose-6-phosphatase; GAPDH, glyceraldehyde 3-phosphate dehydrogenase.

Supplementary Figure 2

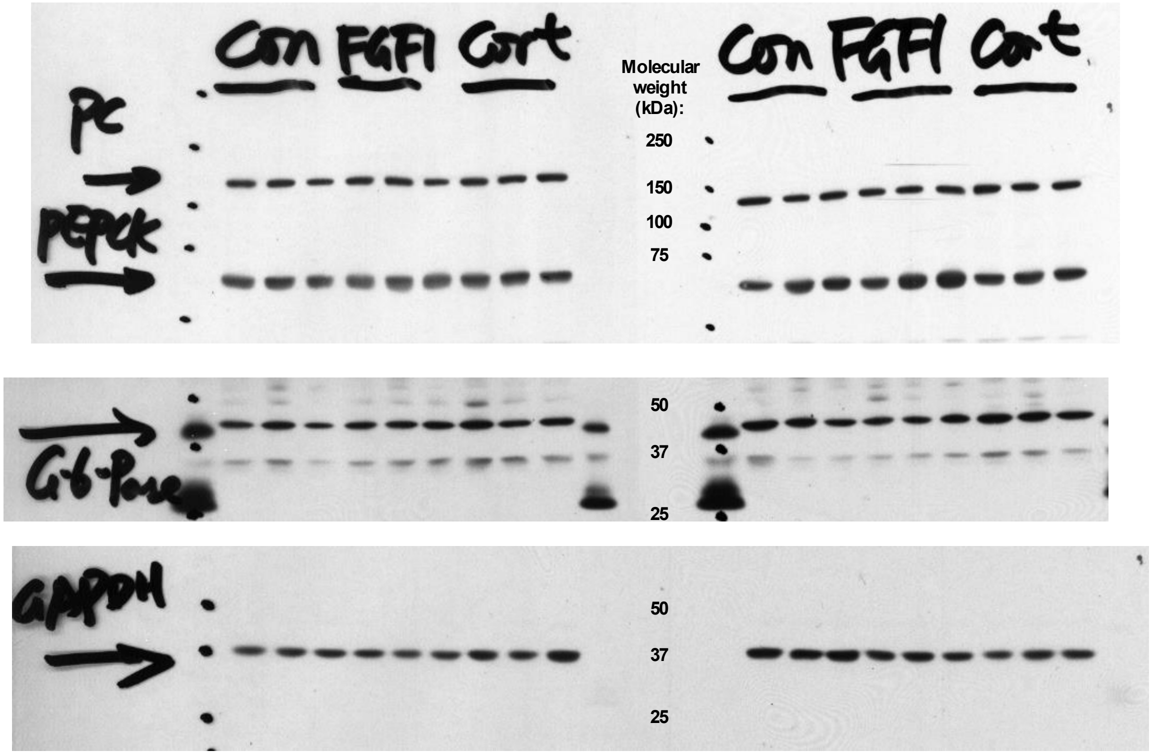

Supplementary Figure 2. Uncut Western blots demonstrating that FGF1 (10 μg) does not affect liver gluconeogenic protein expression. PC, pyruvate carboxylase isoform B; PEPCK, cytosolic phosphoenolpyruvate carboxykinase; G6Pase, glucose-6-phosphatase; GAPDH, glyceraldehyde 3-phosphate dehydrogenase.
